# I Don’t Want to See It End: A Faith Community Nurse Intervention for Older Adults and Caregivers

**DOI:** 10.1093/geroni/igaa057.493

**Published:** 2020-12-16

**Authors:** Loralee Sessanna, Sherry Pomeroy, Yvonne Askew

**Affiliations:** 1 University at Buffalo, Buffalo, New York, United States; 2 Orchard Park Presbyterian Church, Buffalo, New York, United States; 3 Catholic Health Buffalo, New York, Buffalo, New York, United States

## Abstract

The Faith Community Nurse (FCN) provides population-based and other nonclinical services in community settings that are not commonly available to the older adult population. The purpose of the FCN Connections (FCNC) study was to test the impact of the FCNC intervention on the health and well-being of clients (C) age 60 and older with chronic diseases and caregiver (CG) dyads (CCGD) by embedding a FCN in a primary care practice (PCP). A mixed method design was used with two cohorts over 18 months (N = 13 CCGD). Experienced FCNs led the components of the intervention in the CCGD home, including client health assessment, education and support while identifying their caregiver’s perceived needs, providing education and spiritual/emotional support, linking to resources, and connecting the CCGD with the PCP. The CCGD completed measurements at baseline, 6 and 12 weeks. CG knowledge, preparedness, self-efficacy with caregiving activities, and spirituality were measured with statistically significant CG improvement for handling emergencies, making caregiving activities pleasant, and taking care of emotional needs of the client. Spirituality, self-esteem, function, depression, and healthy living domains were measured for clients. At 12 weeks, semi-structured interviews with the CCGD were conducted and transcribed. An experiential/phenomenological framework and Reflexive Thematic Analysis were used to analyze data which generated one overarching theme, ‘I don’t want to see it [study] end’ and key themes, Theme 1 [client]: ‘She was always there to help’ and Theme 2 [caregiver]: ‘It’s like you took the pressure off me for a while’ describing the CCGD’s FCNC experience.

